# Hierarchical model for taxi crashes considering the intrinsic factors of taxi drivers and companies in South Korea

**DOI:** 10.1371/journal.pone.0314743

**Published:** 2025-03-11

**Authors:** Jae-Won Jeon, Joonbeom Lim, Ho-Chul Park

**Affiliations:** 1 Department of Transportation Engineering, Myongji University, Yongin, Republic of Korea; 2 Department of Mobility Policy Research, Korea Transportation Safety Authority, Gimcheon, Republic of Korea; National Institute of Technology Calicut, INDIA

## Abstract

Many studies have been conducted to investigate the diverse human-related factors that contribute to traffic crashes. Human factors have a greater impact on crashes caused by taxi drivers with long driving distances and hours. However, due to issues related to the protection of individual data and the complexity of collecting and processing data, there are limitations in clearly identifying risk factors related to driver characteristics. In this study, we combined in-depth survey data that included characteristics of taxi drivers and the companies they belong to and taxi crash data (2017–2019) in South Korea. However, the combined data showed a high correlation or causality between variables, leading to potential problems, i.e., multicollinearity, hierarchical structure of data, and inefficient analysis. To address this issue, we applied Principal Component Analysis (PCA) to reduce the dimensionality of variables and mitigate the problem. Furthermore, we constructed a hierarchical model considering the hierarchical structure of data in corporate taxis, where drivers are affiliated with specific companies. The analysis revealed that managing fatigue at the company level, managing drivers’ diseases, and other intrinsic factors had a significant influence on Fatal-Injury (FI) crashes. These results indicate that taxi crashes are influenced significantly by both company management factors and driver-related factors. Therefore, policymakers can provide customized preventive measures that consider both aspects.

## Introduction

According to the Traffic Accident Analysis System (TAAS) in Korea, among a total of 50,025 traffic crashes involving commercial vehicles, the 12,224 taxi crashes were the highest proportion of crashes, representing 32.3% of the total number of crashes (as of 2021). Traffic crashes are caused by human factors, vehicles, and roads with human factors being responsible for approximately 90% of the crashes [1]. Traffic crashes involving commercial drivers can be additionally influenced by the managerial practices of commercial vehicle enterprises as well as the risk factors above. [2,3]. In this context, in 2012 the International Organization for Standardization (ISO), in collaboration with the UN and WHO, introduced the International Standard ISO 39001, the Road Traffic Safety Management System for corporate traffic safety management. However, measures to reduce traffic crashes among commercial vehicle drivers in South Korea are similar to those for general drivers. The emphasis is placed on road factors, and improvements are made to make crash-prone roads safer. Vehicle factors are addressed by having commercial vehicle companies equip advanced driving assistance systems (ADAS) and conduct inspections. Regarding human factors, when commercial drivers are employed they must submit the results of a health examination, and digital tachographs are used to record risky driving behavior; however, their actual utilization is minimal in South Korea.

Due to specific human factors, commercial drivers have a higher incidence of traffic crashes than general drivers. For example, taxi drivers and other commercial drivers are prone to aggressive driving behavior, such as speeding and reckless driving, because they are influenced by the impact of such driving on their earnings [4]. In addition, they tend to have excessive confidence in their driving skills due to their familiarity with frequently traveled routes [5]. Furthermore, associations have been found between social-labor variables, such as job stress among commercial drivers (taxi, urban, and intercity bus drivers [6,7]), job insecurity [8,9], social support [10], and driving performance indicators, such as traffic crashes and fines [11]. Taxi drivers work in extremely stressful conditions that can increase the risk of chronic pain, cancer, cardiovascular diseases, and stroke [12–15]. They work long hours, alternate between day and night shifts, and have irregular schedules [16], which can lead to disorders associated with shift work such as circadian rhythm disorders, unidentified syndrome, daytime sleepiness, and performance impairments [17,18]. With their long working hours, high occupational stress, and long-sitting pattern, taxi drivers are exposed to increased health risks [19]. Due to these inherent factors, taxi drivers also are frequently involved with traffic crashes. Considering their extensive driving experience, they play a crucial role compared to road and vehicle factors. However, while the relationship between intrinsic factors and traffic crashes has been investigated, the primary focus has been on sleep disorders [20–22]. In South Korea, commercial drivers, i.e., taxi, bus, and truck drivers, have been found to have up to five times higher probability of sleep disorders due to shift work compared to day-only workers [23]. Also, taxi drivers (transport workers) are influenced significantly by various factors that are known to contribute to traffic crashes, such as driving while drowsy, not paying sufficient attention to the presence of other vehicles, traffic violations, and aggressive driving behavior [24]. Previous studies have indicated that traffic crashes involving commercial drivers are more influenced by the drivers’ intrinsic factors (health, stress, etc.) and whether commercial vehicle companies manage these intrinsic factors sufficiently rather than physical factors (road and vehicle).

In this study, we aim to define and identify the “intrinsic crash factors,” which are distinctly different from the external demographic characteristics of drivers collected in the existing driver management system. These intrinsic crash factors encompass various personal attributes related to the driver’s lifestyle, health, working conditions, emotions, and personality traits, which may influence their driving behavior while operating commercial vehicles. Understanding these intrinsic crash factors was crucial for investigating the fundamental human-related causes of traffic crashes. By analyzing the relationship between these factors and crashes, policymakers can provide customized preventive measures that consider the characteristics of both drivers and transportation companies. However, there were limitations in reflecting the driver’s intrinsic indicators by using traditional traffic crash data or the Digital Tacho Graph (DTG). Therefore, building a model that accounts for these intrinsic factors was essential, and factor analysis techniques would be utilized to address this issue.

Human factors pose challenges when it comes to the collection and analysis of data. These factors are difficult to quantify and may require specific conditions for the collection of data. In addition, issues related to privacy and individual information can arise when attempting to understand the characteristics of the driver in every crash. Due to these challenges, relatively few studies have been successful in the attempt to link human factors to the analysis of crashes. To overcome these obstacles, data collection and surveys at the level of the socio-economic indicators may be necessary. Human factor data tend to exhibit significant correlations and causality between variables, making it suitable for grouping and analyzing variables based on their common characteristics. Utilizing factor analysis helps to reduce the dimensionality of variables, managing the analysis and interpretation easy.

Furthermore, conducting regression analysis on raw data that encompasses human factors can be limited and challenging in constructing models and explaining variables. Without employing predefined procedures, performing regression analysis can lead to challenges such as increased complexity and time consumption due to the potentially high dimensionality of variables in the dataset, as well as the emergence of multicollinearity problems when independent variables are highly correlated. Factor analysis provides a way to address these issues and allows for the grouping of variables that have similar characteristics. The results of factor analysis indicated that multiple latent factors exist within the variables, and statistical grouping was possible at a certain level. In addition, factor analysis confirmed that variables within each group contribute to explaining the variance. Thus, by using factor analysis, researchers can gain insights into the underlying human factors and their impact on traffic crashes, and this will lead to more effective and targeted strategies for preventing crashes.

Taxi crashes exhibit distinct characteristics that may vary based on the management practices of affiliated companies responsible for overseeing taxi drivers. Moreover, the data utilized in this study were collected through surveys, encompassing intrinsic factors related to individual drivers’ personality traits and tendencies. The hierarchical structure of these data allows for distinguishing between different levels, wherein a correlation between traffic crashes can potentially exist between the upper-level entity (company) and the lower-level entity (drivers). This suggests the possibility of correlations based on company affiliation for taxi-related traffic crashes, necessitating consideration for robust modeling of traffic crashes.

The purpose of this study was to analyze the risk factors associated with corporate taxi crashes, focusing on the intrinsic human factors of drivers. Specifically, this research aims to statistically validate the impact of intrinsic human factors on crashes of corporate taxi through a hierarchical model. To collect intrinsic human factors of drivers, extensive surveys were conducted in addition to utilizing existing crash history data. Data preprocessing and variable transformation methods were proposed. Furthermore, the Bayesian hierarchical probit regression analysis model was applied, which considers the hierarchical structure between drivers and the companies for which they work. Through the model, identified crash risk factors were examined thoroughly, and implications for future prevention of corporate taxi crashes were presented, highlighting the significance of this research.

The key contributions of the study can be summarized as follows: (a) This study integrated factor analysis and hierarchical regression model in the field of traffic safety to model the complex and hierarchical relationship between taxi companies, drivers, and taxi crashes; (b) We collected and analyzed data on the health, habits, and company conditions of professional drivers, which had previously been challenging to obtain in existing research; (c) This study provides implications based on significant risk factors identified through the model.

This paper is structured as follows. In literature review, we review existing research on the intrinsic factors of corporate taxi crashes and discuss studies using hierarchical models to investigate traffic crashes. Materials and methods explains the collection and preprocessing of data that were used in this study, and it provides an overview of the Bayesian hierarchical probit regression analysis model. Data preparation includes the process of data manipulation and re-coding of the survey data using Principal Component Analysis (PCA) for statistical model utilization. In results and discussion, we present the results and interpretation of the PCA and Bayesian hierarchical probit model estimation according to the analytical methodology. The paper is concluded in conclusions, which includes a discussion of future research directions.

## Literature review

In this study, we used factor analysis to identify various and numerous personal characteristics of drivers and to analyze the intrinsic factors that contribute to taxi crashes within a hierarchical traffic crash model. Previous research has been conducted on the intrinsic factors associated with corporate taxi crashes and hierarchical traffic crash models. Through a review of relevant prior studies, we have demonstrated the significance and distinctiveness of our research.

### Factor analysis

Factor analysis has been used extensively in various fields, including psychology, education, and sociology, to analyze individual or group characteristics based on the data collected by various surveys [25–27]. In survey-based or various data-driven research, it is essential to verify whether each data item effectively explains the factors that the researcher intends to investigate. In other words, it is crucial to statistically examine the relationship between the survey items and the factors that researchers want to explain. For example, in this study, we aimed to confirm which factors in the daily lives of taxi drivers correspond to survey items and determine whether these factors have an impact on the likelihood of taxi crashes. Through this process, the insights gained from the survey results can be justified. Additionally, factor analysis can be particularly helpful in situations where there are high correlations among the observed variables. In parametric models, multicollinearity can lead to unstable parameter estimates and make it challenging to identify the unique contributions of each variables. Factor analysis helps address this issue by creating orthogonal (uncorrelated) factors, reducing the interdependency among variables and providing more reliable estimates.

Factor analysis has been used in the field of education to evaluate the satisfaction of classes or identify factors that influence the quality of lectures [26,28–30]. Similarly, in the field of public health, numerous studies have utilized factor analysis to examine the correlations between personal characteristics, such as age, gender, race, lifestyle habits, and brain or cardiovascular diseases [31–33]. The factor analysis, e.g., principal component analysis, allows them to condense multiple observed variables into a smaller set of factors that capture the essential information from the data. In these various fields, factor analysis is also used to reduce the dimensions of unquantified data and devise efficient alternatives.

In the field of traffic safety, as in other disciplines, some studies have utilized factor analysis. Syed and Khan [25] and Susilawati and Nilakusmawati [34] used factor analysis to examine socio-economic indicators and general conditions of commercial vehicles. Indeed, they identified the factors that impact mobility and safety of public bus transportation service in order to enhance the efficiency of public transportation operations. In addition, Ma et al. [35] and Zhang et al. [36] used factor analysis based on commonly available data, such as gender, age, driving experience, and the type of vehicle, to investigate risk factors in traffic safety. However, variables that capture the actual drivers’ characteristics or living environment were not included in these studies.

Taxi crashes, the focus of this study, have various intrinsic risk factors of occupational drivers. A detailed analysis incorporating data from multiple sources, including surveys (the primary method for collecting information on the inherent characteristics of drivers and their companies), is required to obtain practically meaningful results. Therefore, factor analysis is needed to analyze these datasets accurately and efficiently.

### Hierarchical regression model

Extensive studies have been conducted to identify the risk factors that influence traffic crashes. However, traditional regression models have limitations in accurately identifying the various factors that impact traffic crashes. In order to derive unbiased estimation results through traditional regression models, the analysis data must satisfy the basic assumptions of the model. However, traffic crash data have characteristics that make it difficult to maintain the independence of residuals, which is one of these basic assumptions [37]. To ensure the independence of residuals, it is necessary to avoid any correlation among the individual traffic crashes under analysis. Nevertheless, real-world situations often exhibit correlations among individual traffic crashes occurring in the same vehicle, on the same road, or within the same area. Traffic crash data with such characteristics can be considered as data with a hierarchical structure, requiring the application of models that consider within-group correlations, with the same road or area treated as the upper-level group.

Crashes involving commercial vehicles exhibit a hierarchical structure and differ from general traffic crashes. In this regard, Park et al. [38] constructed a hierarchical model considering the correlation between individual crashes based on the transportation company for taxis, buses, and cargo vehicles. Using data from 86,622 commercial vehicle traffic crashes over five years (2010-2014), a Cross-Classified Multi-Level Model (CCMM) was developed, taking into account the influence of the company and regional heterogeneity. As a result, the model’s goodness-of-fit improved, enabling more precise identification of the causes of traffic crashes. Kim et al. [39] constructed a hierarchical model, focusing on the high fatality rate of pedestrian crashes in Korea using three years of data (2011-2013). The study identified that not only the micro-level characteristics of pedestrian crashes but also factors at the municipal level, such as population density, proportion of elderly population, and fiscal autonomy, influenced the occurrence of pedestrian crashes. It is a reasonable approach to apply a hierarchical regression model to traffic crash data with such a hierarchical structure.

An attempt has been made to elucidate the intrinsic factors of corporate taxi crashes through the construction of a hierarchical model using survey-based data [40]. However, due to the endeavour to explain multiple factors within a single model, challenges related to the correlation and causality among them were encountered. This can lead to undisclosed type I or type II errors, highlighting the need to control for the characteristics of these factors. Tanglai et al. [41] analyzed the influence of personality and attitude on risky driving behavior among the drivers of public vans. However, studies examining the intrinsic factors of public vehicle drivers, similar to the present study, are scarce.

Additionally, considering not only crash-related data but also a variety of variables including the personal attributes of transportation workers necessitates a re-evaluation of methodologies to overcome the uncertainty associated with these variables [42,43]. This comprehensive approach aims to enhance the accuracy and reliability of the analysis by addressing potential variability and ensuring robust outcomes in the study of transportation safety.

In this study, we deal with hierarchical data that includes various intrinsic risk factors. As mentioned earlier, taxi crashes involving taxi drivers are influenced not only by individual drivers but also by the company they belong to. Specifically, the occurrence of crashes varies depending on factors such as the company’s working conditions and the level of safety management systems. Additionally, important factors include the individual driver’s education level, income, driving distance, working days, and personality traits. To account for this hierarchical structure, we applied hierarchical modeling in this study. Hierarchical modeling allows us to consider the influence of both individual drivers and the companies they work for on the occurrence of crashes. By using hierarchical modeling, we can better understand the complex interactions and relationships between various factors and assess their impact on crash likelihood.

## Materials and methods

### Research process

In this study, variable selection was conducted based on a dataset obtained through a survey administered by a public institution, i.e., the Korea Transportation Safety Authority. The selected variables exhibited high correlations or causalities among factors, necessitating dimension reduction. Principal Component Analysis (PCA), a technique in factor analysis, was used to reduce the dimensionality of the variables. By parameterization, the characteristics of each factor were identified. The data used in this analysis pertained to taxi drivers in the corporate taxi category, allowing for hierarchical differentiation due to the nature of the dataset being specific to individual companies. Using the derived hierarchical and differentiated new variables, modeling was conducted through Bayesian hierarchical probit regression with the occurrence of FI crashes as the dependent variable. The resulting model identified the risk factors associated with taxi drivers based on their respective companies, and the goodness-of-fit of the model was verified through the examination of Deviance Information Criterion (DIC) and Intra-Class Correlation (ICC) values.

### Principal component analysis

In this study, factor analysis was used as a statistical technique to explore and explain the variability among correlated variables, which includes both quantitatively observed data and variables that are inherently difficult to quantify. Factors, by definition, represent latent variables with relatively fewer observable measures compared to the collected quantitative data. Factor analysis was used primarily for explanatory purposes, i.e., to identify and incorporate these latent factors into models. It can be categorized into exploratory factor analysis (EFA), confirmatory factor analysis (CFA), and correlation analysis (CA), each of which are suitable for different research objectives. EFA is used when survey data are available, allowing for the exploration of underlying factors when the certainty of the information is limited for variables with surface-level observations. Alternatively, it is also used when currently there is no statistical or theoretically established framework.

In this study, we used the EFA to identify latent factors using two distinct approaches, i.e., PCA and Common Factor Analysis. For the purpose of this research, the focus was placed on utilizing PCA. Through factor rotation, the exploration of the underlying factors was conducted, allowing for a clear understanding of how variables are relatively heavily loaded onto specific factors. In addition, the computation of inter-variable correlations facilitated the assessment of factor loadings, contributing to the determination of variables’ associations with the identified factors. The Kaiser-Meyer-Olkin (KMO) Measure, Bartlett’s test of sphericity, and communalities were computed, enabling the establishment of factor interpretability, while factor loadings provided valuable insights into the explanatory power of the identified factors.

The KMO Measure is an index that is used to assess the extent to which the correlations among variables can be explained adequately by other variables. Generally, a KMO value of 0.7 or higher is considered acceptable for factor analysis [44]. Bartlett’s test of sphericity is a metric that is used to evaluate the goodness-of-fit of the exploratory factor analysis model. The threshold for the significance level and the acceptance of the p-value vary depending on the desired level of confidence, but typically, a significance level of 0.05 within a 95% confidence interval is used as a criterion [45]. Communality refers to the proportion of variance in a variable that is accounted for by the extracted factors during the analysis. Variables with communality values below 0.4 generally are considered to have low common variance, and therefore they are excluded from the analysis [46]. After factor rotation, factor loadings can be computed from the rotated component matrix. These loadings serve as indicators for determining the classification of each factor. Usually, factor loadings of 0.4 or higher are considered significant, and when the rotated sum of squares loading is higher for a particular factor, the corresponding variables are classified under that factor [47].

The determination of the number of factors in Factor Analysis was based on the cumulative proportion of variance and eigenvalues derived from the analysis. Eigenvalues represent the sum of squared loadings for each factor and serve as an indicator of the explanatory power of a particular factor [48]. Eigenvalues were considered with respect to a threshold value of 1.0. Factors that yielded eigenvalues equal to or greater than 1.0 were retained for further analysis and grouping purposes. These factors were deemed to possess sufficient explanatory capacity to be considered meaningful in the study [49].

### Bayesian hierarchical probit regression

**Fig 1:**
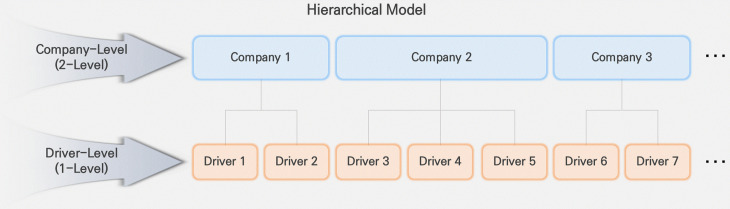
Hierarchical structure of corporate taxi crashes.

Bayesian hierarchical probit regression is a methodology that commonly is used in analyzing traffic crashes, as shown in Fig 1. It is a suitable approach for analyzing data with a hierarchical structure [50]. In this study, the hierarchical model was structured with the upper level representing the taxi companies and the lower level representing individual drivers.

The limitation of traditional probit regression models is that they do not account for the correlation between lower-level crashes of drivers based on upper-level (i.e., corporate taxi company) factors in the hierarchical data analysis. If the regression model doesn’t account for the correlation between crashes across different companies, it could result in underestimating the standard errors of the coefficient values for the independent variables [51,52].


$$\begin{array}{c} \mathrm{Pr}\left(y_{ij}=1\text{|}x_{ij}\right)=\Phi \left(\beta_{0}+\beta_{1}x_{ij}+u_{i}+\varepsilon_{ij}\right) \end{array}$$ (1)

In this equation, $\mathrm{Pr}\left(y_{ij}=1\text{|}x_{ij}\right)$ is the probability that the binary outcome variable, $y_{ij}$, takes the value of 1 for the jth observation within the ith group, given the predictor variable $x_{ij}$. *Φ* denotes the cumulative distribution function of the standard normal distribution. The parameters of interest to estimate are $\beta_{0}$ and $\beta_{1}$, which represent the fixed effects associated with the predictor variables. The term $u_{i}$ represents the random effects at the company level, capturing the unobserved heterogeneity between groups. Finally, $\varepsilon_{ij}$ represents the residual error term. Estimating the hierarchical probit model typically involves using advanced statistical techniques, such as maximum likelihood estimation or Bayesian methods, due to the complexity of the model. These estimation methods account for the correlation within groups and provide estimates of the fixed and random effects [53–55].

In order to conduct Bayesian hierarchical regression, an assessment of the model’s applicability was necessary, and it was performed using the ICC. The ICC indicates the proportion of variance accounted for by the upper level compared to the lower level, and this allows us to assess the extent to which lower-level crashes are influenced by upper-level factors. A high ICC value indicates a strong influence of the upper-level factors. Typically, a threshold of 5% or higher is used to determine the applicability of the model [51].


ICC=σu2σu2+π3 (2)

In the Bayesian paradigm, similar to the probit used in this study, various measures, such as Bayesian coefficients, the Bayesian Information Criterion (BIC), and the Watanabe-Akaike Information Criterion (WAIC) are used to evaluate and compare the performance of fitted models [56]. In this study, the DIC is employed as an indicator to evaluate model fit. A lower DIC value signifies a better model fit [57].


$$\begin{array}{c} DIC=p_{D}+\bar{D\left(\theta \right)} \end{array}$$ (3)

Where $\bar{D}$ represents an estimation of fit, and a reduction in its value indicates an increase in log-likelihood, thereby suggesting an improved model fit. The $p_{D}$ serves as a penalty for the complexity of the model, and this ensures that there is a fair comparison between competing models with different degrees of complexity [58]. In this study, the Markov Chain Monte Carlo (MCMC) method was used for model estimation, which enables the calculation of the DIC.

### Data preparation

This study focuses on the analysis of traffic crashes using data collected over the three years from 2017 to 2019. The study aims to explore the factors that contribute to traffic crashes and their implications for road safety. A survey was conducted to gather the relevant information needed to construct the variables for the analysis. Crash data has been integrated with various survey-derived variables of taxi drivers to establish a dataset that confirms whether intrinsic driver-related characteristics affect the risk of crashes. The data utilized in the analysis consisted of crash data and survey data from corporate taxi drivers, which were matched within the Korea Transportation Safety Authority system to form a unified dataset.

#### Corporate taxi crash data.

In South Korea, crash-related data for corporate taxi drivers is managed and operated through the Traffic Safety Information Management System or the Taxi Operation Information Management System (TIMS). This data is collected with a high level of encryption to ensure the privacy of personal information, and for the purpose of this study, raw data from this source was utilized for crash analysis.

In pursuit of understanding the factors influencing FI crashes among corporate taxi drivers, a comprehensive dataset was compiled. This dataset drew from the records of 63 corporate taxi companies and encompassed information from 1,060 drivers. The dataset utilized for the analysis in this study consisted of individual taxi drivers as the primary units and incorporated both personal information and crash data specific to each driver. It is noteworthy that a significant majority, precisely 59 out of the total corporate taxi companies, had documented instances of FI crashes during the study period. Among the cohort of drivers examined, 484 individuals, constituting 45.66% of the sample, reported their direct involvement in traffic crashes. Strikingly, an important revelation emerged: all drivers who had experienced crashes had invariably been exposed to incidents characterized by a high degree of severity. This finding accentuates the urgency of developing and implementing strategies aimed at mitigating crash severity specifically among taxi drivers.

#### Fusion of crash data and survey data.

This study was focused on conducting an analysis of human factors, specifically the “Black Box,” that contribute to traffic crashes by surveying domestic transport workers in the transportation industry. Through the survey, data were collected and categorized into five distinct characteristics (Table 1), i.e., 1) the working environment, reflecting direct responses from transport workers during their work shifts; 2) the characteristics of the company, which encompasses system and education-related aspects based on the safety management criteria assigned to individual transport workers; 3) living conditions associated with the transport workers’ living environments; 4) physical condition, pertaining to individual’s health status; and 5) the driver’s characteristics, encompassing personal traits and passenger interactions.

**Table 1:** Data description for company and driver levels.

Index	Company Level		Driver Level			Total
**By Type**	Working Environment	Characteristics of the Company	Living Conditions	Physical Conditions	Driver’s Characteristics	
**Number of variables before PCA**	13	11	10	19	9	62
**Number of variables after PCA**	5	5	5	9	5	29

The data were collected based on a survey of 1,060 participants, and the analysis consisted of a total of 62 factors, which were categorized separately based on five selected characteristics and the level of the data. Then, these variables were restructured and reduced to 29 factors through dimension reduction, resulting in a reduction of 53.23%. The correlation among all variables was taken into consideration during the restructuring process. For the restructure, PCA was conducted to determine the commonality among the independent variables and to calculate the factor loadings. Exploratory factor analysis was performed using varimax rotation to extract the explanatory variables and to reduce the dimensionality of the data further. The values of the grouped variables were recalculated and used as independent variables in the Bayesian hierarchical probit model. The crash occurrence of taxi driver used as the dependent variables were collected and managed by the Korea Transportation Safety Authority, and the data were matched to all participants who responded to the survey.

## Results and discussion

### Principal component analysis

A survey was conducted and the data from system collection items were utilized as variables among 1,060 taxi drivers. The variables were grouped as presented in Table 2, and the component matrix and interpretation according to each variable as shown in Table 3.

**Table 2:** Output of principal component analysis for the company level.

Factor No.	Input Variables	*λ*	Output Variables
**1**	Overall job satisfaction	0.835	Job satisfaction
	Satisfaction with wage level	0.814	
	Satisfaction with welfare benefits	0.803	
	Satisfaction with working hours	0.801	
**2**	Management of lack of sleep	0.785	Driver fatigue management by the company
	Managing the driving hours	0.785	
	Management of drivers’ breaks	0.765	
	Management of the psychological status	0.735	
**3**	Working two shifts a day	−0.960	Each person owns a car
	Each person owns a car	0.969	
**4**	Flat payment system	−0.938	Payment system
	Full management system	0.927	
**5**	The physical burden of drivers by the company	0.868	Degree of the physical burden by the company
**6**	Lack of safe driving education is the main cause of the crash	−0.951	Degree of interest in company drivers
	Reports of violence from passengers	0.943	
**7**	Management of personal diseases	−0.897	Driver crashes and disease management
	Personal crash management	−0.894	
**8**	Number of training sessions per year	0.786	Degree in company-level education
	Management’s perception of support for The crash reduction of transportation companies	0.746	
	Crash prevention education	0.548	
**9**	Safety training for elderly drivers	−0.773	Degree of education related to up-to-date laws
	Education of revision of the relevant statutes (Road Traffic Act, Transportation Commercial Act)	0.768	
**10**	Recommendation and training for compliance with break times	0.866	Relaxation/health programs and education
	Implementation of a healthcare support program	0.656	

**Table 3:** Output of principal component analysis for driver level.

Factor No.	Input Variables	*λ*	Output Variables
**1**	Divorce or bereavement	0.942	Personal characteristics of living space
	Living alone	0.942	
**2**	The amount of alcohol drunk on a day off	0.936	Usual drinking habits
	Frequency of drinking	0.928	
**3**	Leisure activities_sports	0.797	Active leisure activities
	Leisure activities_hobbies and recreational activities	0.797	
**4**	The main cause of crashes due to irregular life	0.733	Irregular personal life
	The main cause of crashes due to driving while drowsy	0.676	
**5**	Measure and manage whether drivers are drinking alcohol	−0.688	Social relationship
	Satisfaction of colleague relationship	−0.672	
**6**	Health management effect of specialized precision health checkup	0.978	Driver-specific precision health checkup
	Specialized precision health checkup experience for drivers	0.978	
**7**	Cardiovascular (circulatory) diseases	0.764	Cardiovascular/Endocrine disease
	Endocrine disease	0.724	
	Management of individual diseases of drivers	0.596	
**8**	The need to establish a national fatigue management system	−0.885	Importance of drivers’ healthcare
	The main cause of crashes due to drivers’ health	−0.762	
**9**	The need to develop and disseminate self-diagnosis techniques for diagnosing fatigue	0.901	The need for institutional methods for health
	The need for system improvement related to working conditions	0.800	
**10**	The need for the management of drivers’ fatigue	0.866	The need for company-sponsored healthcare
	Driver’s disease inspection and prevention education	0.828	
**11**	Digestive system disease	0.736	Digestive system/Kidney and urological diseases
	Kidney and urological diseases	0.696	
**12**	Respiratory system disease	0.747	Respiratory system/Surgical diseases
	Surgical-related diseases	0.648	
**13**	Need for development and dissemination of fatigue measuring equipment	−0.940	The need for equipment to measure fatigue
**14**	Medication for other than chronic disease	0.719	Interest in healthcare
	Regular health care	−0.637	
	Cerebrovascular disease	0.453	
**15**	Strengthening punishment by law as a measure to eradicate violence from passengers	−0.855	The necessity of preventing violence among passengers through social institutions
	Raising social awareness as a way to eradicate violence from passengers	−0.807	
**16**	The main cause of crashes due to drivers’ violations	0.842	The main cause of crashes due to company deposit
	The main cause of crashes due to the burden of company deposits	0.817	
**17**	The main cause of crashes due to the burden of company deposits	0.787	Job stress due to physical/mental fatigue
	Crash vehicle management level	0.670	
	Job stress-inducing factors_physical Fatigue	0.551	
**18**	Job stress-inducing factors_work environment always exposed to crash	−0.944	Job stress resulting from the environment exposed to the Crash
**19**	Effect of education being conducted	−0.872	Effect of education being conducted

The PCA analysis was performed on input variables representing various types of basic states. The output variables were derived based on a loading threshold of 0.4, indicating the variables that showed significant influence on the data. All of the variables were grouped and interpreted effectively, undergoing the reparameterize process. Through this process, each variable was appropriately named according to its characteristics, resulting in the reconfiguration of the dataset for regression analysis. This restructured dataset serves as the input data for the Bayesian hierarchical probit model, with working environment and characteristics of company variables designated as the upper-level variables in the hierarchy, representing the company-related aspects.

When reparameterized from the original raw data, some variables may have loading values that are either negative or a mix of positive and negative values. In such cases, the interpretation of the reparameterized variables can differ from the original data’s meaning. For instance, the variable “each person owns a car” includes both a (−) value “working two shifts a day” and a (+) value “each person owns a car,” typically representing situations where respondents can only provide opposing responses to the original variable. Therefore, in such instances, one of the variables is selected for building the regression model. Another example is “social relationship,” where all included input variables have negative loadings, resulting in reparameterized variables with opposite meanings. Such cases offer various interpretations based on the characteristics of the loading values derived from PCA, and all possibilities were considered when utilized in the analysis.

### Bayesian hierarchical probit regression

We developed a hierarchical traffic crash model for corporate taxis using a selected set of independent variables from the hypothesis testing stage, and the occurrence of crashes among taxi drivers in the past three years as the dependent variable. To assess the applicability of the Bayesian hierarchical probit model, we calculated the ICC, resulting in a value of 33.98% (Table 4). This result indicated that the construction of a hierarchical crash model is appropriate. The ICC value implies a substantial variability in individual taxi driver crash rates at the lower level, which is attributed to differences among upper-level taxi companies. In other words, the occurrence of crashes among taxi drivers varies significantly depending on the taxi company they belong to. A hierarchical model is necessary to identify the factors that influence this variability. Therefore, our findings demonstrate the necessity of a hierarchical approach in exploring the factors that contribute to the variation in crash occurrence among taxi drivers based on their affiliations with different taxi companies.

**Table 4:** Performance of the Bayesian hierarchical probit Model.

Estimation Result for Variations & ICC		
Index	Value	S.E.
Company-Level $\sigma_{v_{0j}}^{2}$	0.539	0.149
ICC	33.98%	
**Model Performance Results**		
DIC	Logit Model	Probit Model
Traditional Model	1463.893	1463.866
Bayesian Hierarchical Model	1293.724	**1291.248**

To construct the regression models, we examined the significance level of each variable, and those variables that did not reach a statistical significance level of 90% based on the 0.1 criterion were excluded from the model. To prevent multicollinearity issues among the variables, we assessed the Variance Inflation Factor (VIF) and ensured it did not exceed 10 [59].

After constructing the Bayesian hierarchical probit model, we set the dependent and independent variables identically and built comparative models (i.e., conventional logit and probit models and Bayesian hierarchical logit model). As a result, the DIC, which is used to assess the fit of the model, shows that 1291.248 for the Bayesian hierarchical probit model, 1293.724 for the Bayesian hierarchical logit model, and 1,463.893 for the general logit model (Table 4). This indicates that the Bayesian hierarchical probit model demonstrates a better fit compared to the conventional and Bayesian hierarchical logistic model.

As shown in Table 5, in the analysis at the company level (2-level), it was observed that the probability of traffic crashes decreases as the management of drivers’ fatigue levels are increased by the company. This factor includes management of lack of sleep, managing the driving hours, management of drivers’ breaks, and management of the psychological status. This finding supports the rationale that fatigue management at the company level is crucial, and it highlights the need for a more systematic approach to ensure the traffic safety of the drivers. Specifically, the factor “the need for fatigue measuring equipment” emerged as a significant factor at the driver level (1-level), indicating that establishing a fatigue management system within the company can effectively reduce traffic crashes [60–62].

**Table 5:** Estimation results of the Bayesian hierarchical probit Model.

Bayesian Hierarchical Probit Model					
Level	Variables	β	S.E.	p−value	VIF
2-Level (Company Level)	Driver fatigue management by the company	−0.103	0.043	0.010^***^	3.391
	Each person owns a car	−0.121	0.044	0.002^***^	1.140
	Drivers’ crashes and disease management	0.314	0.057	0.000^***^	3.593
1-Level (Driver Level)	Social relationship	0.013	0.003	0.000^***^	2.998
	Importance of drivers’ healthcare	0.291	0.077	0.000^***^	1.322
	Digestive system/Kidney and urological diseases	0.195	0.132	0.068^**^	1.480
	The need for fatigue measuring equipment	−0.230	0.083	0.002^***^	2.206
	The necessity of preventing passenger violence through social institutions	−0.101	0.060	0.049^**^	1.317

*** p <  0.01, ^**^ p <  0.05, ^* ^ p <  0.1.

The next noteworthy result pertains to disease-related factors. These factors are manifested as “driver crashes and disease management” at the upper-level, and at the lower- level, they include “importance of driver’s health care” and “digestive system/Kidney and urological diseases.” In fact, the presence of diseases among drivers is associated with other conditions, leading to negative synergistic effects [63]. Various studies have indicated that these diseases, as well as the medications taken for them, can have a direct or indirect correlation with traffic crashes [64,65]. Therefore, it can be observed that it is possible to mitigate the occurrence of traffic crashes by implementing health management at both the company and individual levels [66].

In addition, various intrinsic factors such as “social relationship” was found to be included in the analysis results, increasing the probability of traffic crashes, while “each person owns a car” and “the necessity of preventing passenger violence” were found to decrease it. The variable “each person owns a car” was reparameterized from the raw data as a variable related to the rotational shift pattern at the company level, and it is included as a 2-level variable. Research findings suggest that crashes involving taxi drivers who do not own the vehicle they operate have a higher likelihood of engaging in risky driving behavior compared to those who own their vehicles [67]. This indicates a close relationship between intrinsic factors, such as moral laxity and risky driving behavior. Some studies also have revealed a significant correlation between the social relationships of drivers and the occurrence of traffic crashes, highlighting the need for targeted management for specific drivers [65]. Furthermore, research has consistently shown that traffic crashes resulting from passenger types and behaviors are prevalent, emphasizing the importance of company-level education and regulations implemented by government or public institutions to mitigate the probability of taxi-related crashes caused by such issues.

### Implications for preventing taxi crashes

Regarding the results of model construction, at the company level, taxi crashes (excluding personal taxi crashes) were found to be influenced by the management of drivers by the transportation company. In addition, at the driver level, it was found that the more importance the driver placed on health management and the need for fatigue measurement equipment, the more negative impact it had on traffic crashes. In Korea, commercial vehicle companies have the authority to manage affiliated drivers, but only a few companies manage fatigue or health. As long as it does not violate the Labor Standards Act or the contractual obligations between the commercial vehicle company and the driver, fatigue management for drivers is not strongly emphasized. In the Passenger Transport Service Act in Korea, safety inspections of transportation companies are conducted mainly on vehicles, and there are limitations in checking drivers’ compliance with recommended rest time. Although all commercial vehicles in Korea have a digital tachograph that records driving time, rest time, and risky driving behaviors, it is not utilized to punish drivers or commercial companies. However, it can be used by government agencies or transportation companies for driver education purposes.

Nevertheless, transportation companies do not actively educate or check fatigue management for drivers. Research on the objective determination of the correlation between driver fatigue and traffic crashes in Korea is limited, so it has not been firmly established institutionally. In contrast, in the United States, the National Transportation Safety Board (NTSB) revealed that fatigue was the leading cause of 31% of large truck fatal crashes based on the analysis of 182 crashes in 1990. In addition, the Federal Motor Carrier Safety Administration (FMCSA) stated that fatigue was the cause of 13% of severe commercial motor vehicle crashes. As a result, the National American Fatigue Management Program (NAFMP) for commercial motor vehicle drivers has been jointly researched and implemented by transportation companies, drivers, and research institutions in the United States for several years. The program’s manual covers transportation companies’ culture, education, and training, sleep disorder examination and treatment, fatigue monitoring technology, and scheduling. In Korea, legislation regarding fatigue and disease management for drivers by transportation companies should be prioritized, and after legislation, equipment to measure driver fatigue should be developed and disseminated.

Looking at another factor at the driver level from the results of model construction, it was found that drivers with digestive system diseases or kidney-related diseases positively impacted traffic crashes. Since individual medical data are susceptible, in this study, individual drivers’ traffic crash records were matched and analyzed based on surveys rather than individual medical data. Although the data in this study are based on survey results and not individual medical data, they have significant meaning if the reliability of these data is ensured. Taxi drivers work in shifts, work long hours sitting, and face high job-related stress, which can negatively affect health. These health implications can lead to drivers’ inattention, drowsiness, and cardiovascular diseases, and the result is likely to be adverse impacts, including traffic crashes. In particular, in Korea, the number of elderly drivers in commercial taxis is increasing, so it is urgent to check and manage the health status of drivers. There is a case in Queensland, Australia in which, according to the 2011 Queensland Work Health and Safety Act, taxi company operators should guarantee the health and safety their taxi drivers. Taxi company operators must register shift schedules for no more than 12 hours, register for no more than six days per week, and provide adequate rest time. They also must report to the company anything that affects the drivers’ fatigue. In addition, the law mandates that taxi companies install security devices, such as cameras and emergency buttons, to respond to passengers who threaten the safety of taxi drivers. In the analysis results of this study, taxi drivers who responded to the necessity of safety devices for dealing with violent passengers also had reduced traffic crashes.

The Korea Transportation Safety Authority in South Korea currently manages taxi and bus drivers, but the system lacks adequate periodicity and comprehensive management structures. To address the high proportion of traffic crashes involving commercial vehicles such as taxis and buses, it is crucial to integrate the intrinsic factors of transportation workers into the management system. This study highlights the need for collecting and managing accident data across various commercial vehicles, including corporate and individual taxis, buses, and Demand Responsive Transport (DRT) vehicles. By analyzing this data, the specific characteristics of each transportation company can be better understood, leading to more systematic and effective management strategies. Such an approach is expected to significantly reduce traffic crashes involving commercial vehicles and lower overall societal costs.

## Conclusion

Previous studies on corporate taxi crashes have been limited in terms of exploring the crash probability of drivers, and there have been few studies that utilize actual crash data, relying mostly on survey results reflecting individuals’ characteristics. Although some studies have utilized data, they have been limited by considering only simple and general variables. In this study, we used Bayesian hierarchical probit regression to analyze the factors that significantly influence the probability of crashes, taking into account the characteristics of the companies, using a combination of corporate taxi crash data collected over three years (2017–2019) and survey data. In addition, dimension reduction techniques were applied to highly correlated factors to summarize multidimensional data into low-dimensional data efficiently and identify the risk factors associated with the probability of crashes.

The results of the analysis can be summarized into three main categories as follows. First, factors related to driver fatigue management; second, factors related to driver health status and management; and third, other detailed factors, such as vehicle ownership, social relationships, and the presence of social institutions to address passengers’ violence. These research findings indicate that significant efforts are required not only from the perspective of individual drivers but also from the standpoint of the company to manage crashes effectively. Some studies argue that dedicated organizations and separate institutional measures should be established [61,62,66]. Furthermore, the need for social interest and efforts to reduce corporate taxi crashes is emphasized.

A process was established to construct statistical analysis methodology for utilizing the intrinsic variables of individuals based on survey data. However, there are limitations in clearly grouping all variables. To address this, there is a need for pre-emptive operation of systematic institutions and organizations to manage corporate taxis, and survey questions should be designed in line with the objectives of these institutions and organizations. In the future, if the dataset is constructed to include this process, more distinct reparameterization will be possible, and various dependent variables (such as the occurrence of crashes, crash severity, etc.) can be utilized to identify factors influencing traffic crashes.

In future research, it is also deemed necessary to conduct detailed investigations and analyze the data structure to identify clear risk factors for understanding crash causality. The taxi crash is limited to being explained only by intrinsic factors derived from surveys, and a dataset should be constructed to reflect the actual traffic conditions at the time of the crash. Also, results demonstrate similar findings to research conducted in other countries, and some factors are already being implemented as policies in certain countries. Since taxi crashes are influenced by transportation company management factors and driver factors, policies that manage both factors are necessary to expect a reduction in traffic crashes. In the future, it is necessary to conduct additional research based on objective data from drivers and transportation companies, rather than survey-based data, to scientifically prove the intrinsic factors of taxi drivers and management factors of transportation companies.

The causes and severity of accidents involving taxi drivers exhibit significant diversity. Attempting to elucidate individual characteristics through a singular model poses limitations, and this study, in particular, did not incorporate such individual heterogeneity into the model. Although the model employed herein did not explicitly account for the individual heterogeneity inherent to taxi drivers, it enabled the classification of driver groups sharing similar characteristics. The principal objective of this research primarily revolves around offering policy recommendations directed towards the reduction of taxi-related accidents by governmental or safety management authorities. However, it is emphasized that, in light of the introduction of more comprehensive safety management policies, addressing the multifaceted causes and severity of accidents on an individualized basis should be integrated into these overarching policies.

## Appendix

The survey data utilized in this study consisted of 62 items, and the pre-dimensionality reduction form, prior to employing Factor Analysis, is presented in the Table 6 below for

**Table 6:** Basic statistics of raw data utilized for analysis.

No	Variable	Average	S.D.	Max	Min
1	Overall job satisfaction	0.029	0.169	1.000	0.000
2	Satisfaction with wage level	0.008	0.087	1.000	0.000
3	Satisfaction with welfare benefits	0.420	0.494	1.000	0.000
4	Satisfaction with working hours	0.382	0.813	5.000	0.000
5	Management of lack of sleep	1.642	1.007	5.000	1.000
6	Managing the driving hours	1.642	1.007	5.000	1.000
7	Management of drivers’ breaks	1.642	1.007	5.000	1.000
8	Management of the psychological status	1.642	1.007	5.000	1.000
9	Working two shifts a day	2.218	2.218	5.000	1.000
10	Each person owns a car	1.178	2.815	5.000	1.000
11	Flat payment system	0.054	0.226	1.000	0.000
12	Full management system	0.297	0.457	1.000	0.000
13	The physical burden of drivers by the company	1.642	1.007	5.000	1.000
14	Lack of safe driving education is the main cause of the crash	1.642	1.007	5.000	1.000
15	Reports of violence from passengers	1.642	1.007	5.000	1.000
16	Management of personal diseases	0.008	0.087	1.000	0.000
17	Personal crash management	1.642	1.007	5.000	1.000
18	Number of training sessions per year	0.648	0.478	1.000	0.000
19	Management’s perception of support for The crash reduction of transportation companies	1.642	1.007	5.000	1.000
20	Crash prevention education	1.642	1.007	5.000	1.000
21	Safety training for elderly drivers	1.642	1.007	5.000	1.000
22	Education of revision of the relevant statutes (Road Traffic Act, Transportation Business Act)	1.642	1.007	5.000	1.000
23	Recommendation and training for compliance with break times	4.378	0.632	5.000	1.000
24	Implementation of a healthcare support program	1.642	1.007	5.000	1.000
25	Divorce or bereavement	0.029	0.169	1.000	0.000
26	Living alone	0.027	0.163	1.000	0.000
27	The amount of alcohol drunk on a day off	4.083	0.812	5.000	1.000
28	Frequency of drinking	4.258	0.671	5.000	1.000
29	Leisure activities_sports	2.171	0.723	5.000	1.000
30	Leisure activities_hobbies and recreational activities	1.642	1.007	5.000	1.000
31	The main cause of crashes due to irregular life	0.054	0.226	1.000	0.000
32	The main cause of crashes due to driving while drowsy	1.792	2.161	5.000	0.000
33	Measure and manage whether drivers are drinking alcohol	1.642	1.007	5.000	1.000
34	Satisfaction of colleague relationship	1.642	1.007	5.000	1.000
35	Health management effect of specialized precision health checkup	2.633	0.867	5.000	1.000
36	Specialized precision health checkup experience for drivers	2.558	0.871	5.000	1.000
37	Cardiovascular (circulatory) diseases	1.598	1.675	5.000	0.000
38	Endocrine disease	3.302	0.747	5.000	1.000
39	Management of individual diseases of drivers	0.082	0.275	1.000	0.000
40	The need to establish a national fatigue management system	1.642	1.007	5.000	1.000
41	The main cause of crashes due to drivers’ health	1.642	1.007	5.000	1.000
42	The need to develop and disseminate self-diagnosis techniques for diagnosing fatigue	1.642	1.007	5.000	1.000
43	The need for system improvement related to working conditions	1.642	1.007	5.000	1.000
44	The need for the management of drivers’ fatigue	1.642	1.007	5.000	1.000
45	Driver’s disease inspection and prevention education	0.382	0.813	4.000	0.000
46	Digestive system disease	2.066	1.794	5.000	0.000
47	Kidney and urological diseases	0.416	0.493	1.000	0.000
48	Respiratory system disease	0.648	0.478	1.000	0.000
49	Surgical-related diseases	0.014	0.118	1.000	0.000
50	Need for development and dissemination of fatigue measuring equipment	1.642	1.007	5.000	1.000
51	Medication for other than chronic disease	0.007	0.081	1.000	0.000
52	Regular health care	0.420	0.494	1.000	0.000
53	Cerebrovascular disease	0.601	0.490	1.000	0.000
54	Strengthening punishment by law as a measure to eradicate violence from passengers	1.642	1.007	5.000	1.000
55	Raising social awareness as a way to eradicate violence from passengers	0.297	0.457	1.000	0.000
56	The main cause of crashes due to drivers’ violations	1.642	1.007	5.000	1.000
57	The main cause of crashes due to the burden of company deposits	1.642	1.007	5.000	1.000
58	The main cause of crashes due to the burden of company deposits	0.108	0.311	1.000	0.000
59	Crash vehicle management level	3.408	0.870	5.000	1.000
60	Job stress-inducing factors_physical Fatigue	0.060	0.238	1.000	0.000
61	Job stress-inducing factors_work environment always exposed to crash	0.060	0.238	1.000	0.000
62	Effect of education being conducted	0.060	0.238	1.000	0.000
